# Enhancement of Bonding and Mechanical Performance of Epoxy Asphalt Bond Coats with Graphene Nanoplatelets

**DOI:** 10.3390/polym15020412

**Published:** 2023-01-12

**Authors:** Fan Jing, Rui Wang, Ruikang Zhao, Chenxuan Li, Jun Cai, Guowei Ding, Qingjun Wang, Hongfeng Xie

**Affiliations:** 1 MOE Key Laboratory of High Performance Polymer Materials and Technology, School of Chemistry and Chemical Engineering, Nanjing University, Nanjing 210093, China; 2 Department of Chemistry, Texas A&M University, College Station, TX 77843-3255, USA; 3 Public Instrument Center, School of Chemistry and Chemical Engineering, Nanjing University, Nanjing 210023, China

**Keywords:** nanocomposites, epoxy asphalt, graphene nanoplatelets, bond coat, bonding strength, mechanical properties, phase separation

## Abstract

Improving bonding and mechanical strengths is important for the application of bond coats used in the construction of steel deck bridges. Graphene nanoplatelets (GNPs) are attractive nanofillers for polymer modification because of their low cost, ultra-high aspect ratio, and extraordinary thermal and mechanical performance. In this paper, GNPs were used to reinforce the epoxy asphalt bond coat (EABC). The morphology, viscosity–time behavior, contact angle, dynamic mechanical properties, and mechanical and bonding strengths of GNP-reinforced EABCs were investigated using laser confocal microscopy, a Brookfield rotational viscometer, a contact angle meter, dynamic mechanical analysis, a universal test machine, and single-lap shear and pull-off adhesion tests. GNP dispersed non-uniformly in the asphalt phase of EABC. The viscosity of the neat EABC was lowered with the inclusion of GNPs and thus the allowable construction time was extended. The existence of GNPs enhances the hydrophobicity of the neat EABC. When adding more than 0.2% GNP, the storage modulus, crosslinking density and glass transition temperatures of both asphalt and epoxy of the neat EABC increased. The mechanical and bonding properties of the neat EABC were greatly enhanced with the incorporation of GNPs. Furthermore, the mechanical and bonding strengths of the modified EABCs increased with the GNP content. GNP-reinforced EABCs can be utilized in the pavement of long-span steel bridges with long durability.

## 1. Introduction

Graphene has attracted great interest in applications of polymer nanocomposites because of its unique combination of exceptional thermal, mechanical and electrical properties [1,2,3]. The superior performance of graphene is attributed to its single two-dimensional (2D) layer, which is composed of a honeycomb structure and *sp*^2^ carbon atoms. However, single-layer graphene is expensive, and its mass production is hard to achieve as yet [4]. Because of their feasibility when it comes to large-scale production, cheaper derivatives of graphenes, like graphene oxide (GO) and graphene nanoplates (GNPs), have attracted much attention in the field of polymer/graphene nanocomposites [5,6,7,8,9]. GNPs are platelet-like graphite nanocrystals composed of 2–10 layers of graphenes. In addition to low cost, GNPs exhibit other promising properties, including light weight, ultra-high aspect ratio, outstanding toughness, flexibility, thermal stability, and electrical conductivity [10,11,12,13]. Therefore, GNPs have been widely applied in polymer nanocomposites [14,15,16,17,18,19].

Orthotropic steel decks (OSDs) have been extensively utilized in bridge construction all over the world since 1948 thanks to their rapid erection, light weight, low maintenance costs, and high degree of prefabrication and standardization [20]. To protect the deck from corrosion, provide a comfortable and anti-skidding surface on the plate, and compensate for the deformation of the deck, a wearing surface (concrete) has been paved onto the OSD plate. Furthermore, a bonding layer has been employed to adhere the concrete to the deck plate, with the formed composite action being able to withstand heavy traffic loads [21]. The bonding strength of the bond coat should be as high as possible in order to avoid the delamination and shoving of the wearing surface from the plate. Furthermore, the thin OSD plate (12−16 mm) is highly flexible. In this case, the mechanical properties, especially toughness, of the bond coat should also be outstanding. Therefore, thermosetting epoxy-based bond coats have been widely utilized in the construction of steel bridges [22,23,24]. As an abbreviation denoting epoxy-modified asphalt, epoxy asphalt was initially developed for the pavement of airfields in the late 1950s [25]. Due to the presence of crosslinked epoxy networks, epoxy asphalt shows good adhesion, high strength and excellent water and solvent resistance compared to conventional thermoplastic asphalts [26,27,28,29]. Since 1967, epoxy asphalt has been applied extensively as both the bond coat and binder in the pavement of long-span OSD bridges [30,31,32,33,34]. When used as a bonding layer in the OSD system, the epoxy asphalt bond coat (EABC) needs to have high bonding and mechanical strengths. To improve these properties, nanoclays, such as montmorillonite (MMT) and attapulgite (ATT), and GO have been introduced into EABC [35,36]. Although GNPs have been utilized to reinforce both epoxy and asphalt [37,38,39,40,41], as well as epoxy asphalt binders [42,43], less attention has been paid to GNP-reinforced EABCs.

To improve the bonding and mechanical properties of EABC, GNPs were introduced as a nanofiller. To achieve this aim, GNPs were first mixed with base asphalt and curing agent to prepare a GNP-reinforced bituminous curing agent masterbatch. Then, the masterbatch was mixed with the epoxy prepolymer. The viscosity, hydrophobicity, bonding strength, dynamic mechanical properties, mechanical performance, and morphology of GNP-reinforced EABCs were studied. Furthermore, the failure mechanism of bonding properties of GNP-reinforced EABCs was interpreted.

## 2. Materials and Methods

### 2.1. Materials

Graphene nanoplate powders were bought from Suzhou Tanfeng Graphene Technology Co., Ltd. (Suzhou, China). The physical properties of GPNs are summarized in Table 1. Asphalt binder was provided by China Offshore Bitumen (Taizhou) Co., Ltd. (Taizhou, China). Table 2 presents an overview of the asphalt binder. Bisphenol A epoxy prepolymer with an epoxide number of 0.52 mol/100 g was obtained from Nantong Xingchen Synthetic Material Co., Ltd. (Nantong, China). The acid-based curing agent was self-prepared in the laboratory.

### 2.2. Preparation of GNP-Reinforced EABCs

**Figure 1:**
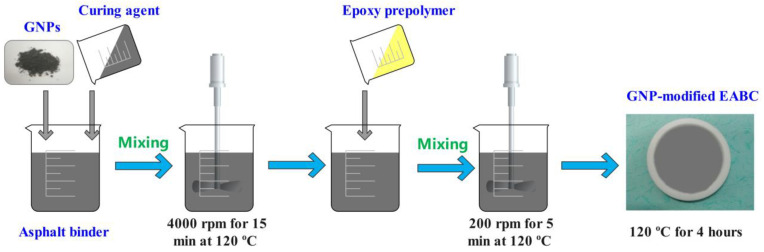
Scheme of the preparation of GNP-reinforced EABC.

Graphene nanoplatelet powders were mixed at 120 °C with the curing agent and base asphalt to prepare a masterbatch using a high-speed emulsifier at 4000 rpm for 15 min. Afterward, the GNP-reinforced bituminous curing agent was mixed with the epoxy prepolymer at the same temperature for five minutes at 200 rpm. Finally, the mixture was poured into a circular Teflon mold with a diameter of 100 mm. The cured GNP-reinforced EABC was removed from the mold after curing at 120 °C for four hours in an oven. A scheme of the preparation of GNP-reinforced EABC is shown in Figure 1. The mass ratio of the epoxy prepolymer, asphalt binder and curing agent is 1:2.5:2. In the modified EABCs, the weight percentages of GNPs are 0, 0.2%, 0.5% and 1%, respectively.

### 2.3. Methods

#### 2.3.1. Viscosity–Time Behavior

The viscosity–time behavior during curing was tested on a Brookfield rotational viscometer (NDJ-1C, Changji, Shanghai, China) under the guideline of ASTM D4402. The uncured GNP-reinforced EABC was transferred into the chamber of the viscometer. The test was conducted at 120 °C using the No. 28 spindle at 50 rpm.

#### 2.3.2. Phase-Separated Microstructures

The dispersion of GNPs in EABCs was observed on a confocal microscope (LSM710, Zeiss, Jena, Germany) with Ar^+^ as the laser source (488 nm). To prepare the sample for confocal microscopy observation, a drop of uncured GNP-reinforced EABC liquid was the slide glass. After covering a cover glass, the glass slide sample was cured at 120 °C for four hours. The distribution of discontinuous asphalt domains in EABCs was determined by an image analysis software. The number- and weight-average diameters (*d_n_* and *d_w_*) and polydispersity index (*PDI*, *d_w_*/*d_n_*) were calculated using Equations (1) and (2):

(1) $$d_{n}=\frac{\Sigma n_{i}d_{i}}{\Sigma n_{i}}$$

(2) $$d_{w}=\frac{\Sigma n_{i}d_{i}{}^{2}}{\Sigma n_{i}d_{i}}$$

where *n_i_* is the number of domains with a diameter of *d_i_*. To determine these parameters, three confocal microscopy images for each sample were used.

#### 2.3.3. Hydrophobicity

The contact angles of GNP-reinforced EABCs were determined using a KSV CAM contact angle meter (Helsinki, Finland). The contact angle was recorded within 60 s after dripping 5 μL deionized water on the sample. At least five measurements were performed for each sample.

#### 2.3.4. Dynamic Mechanical Analysis (DMA)

Dynamic mechanical properties were evaluated on a DMA instrument (DMA + 450, 01 dB-Metravib, Limonest, France). The measurement was performed in tension mode at a heating rate of 3 °C/min and 1 Hz between −50 °C and 100 °C.

#### 2.3.5. Mechanical Performance

Mechanical properties were determined using a universal testing machine (Instron 3366, Instron, Norwood, MA, USA) as per ASTM D638. The dog-bone-shaped samples were measured at room temperature and a crosshead speed of 200 mm/min. The mechanical properties were averaged from five samples.

#### 2.3.6. Shear Strength

The single-lap shear measurements were also performed on a universal testing machine (Instron 3366, Instron, Norwood, MA, USA) as per ASTM D1002. The end surfaces (12.5 mm) of steel plates (100 × 25 × 2 mm^3^) were sandblasted to remove all impurities and corrosion and cleaned with acetone. After coating with the uncured bond coat (12.5 mm), the parts of the two plates were overlapped and cured. Single-lap shear strength was determined at a crosshead speed of 50 mm/min after curing at 120 °C for 4 h and being cooled to room temperature. Five replicates were tested for each sample.

#### 2.3.7. Pull-Off Adhesion Strength

Pull-off adhesion tests were conducted on a DeFelsko PosiTest AT-A portable automatic adhesion tester (Ogdensburg, NY, USA). Q345D steel plates (150 × 150 × 20 mm^3^) were sandblasted and cleaned with acetone. The polished surfaces of steel plates were coated with a layer of uncured bond coat (600 g/m^2^). Afterward, five dollies were secured on the steel plate surface. After curing at 120 °C for 4 h, a core drill was used to separate the testing areas around the dollies. Pull-off adhesion tests were performed using a uniform tensile force on the dolly by using the automatic adhesion tester under a speed of 0.7 MPa/s at room temperature and 60 °C.

## 3. Results and Discussion

### 3.1. Phase Separation

**Figure 2:**
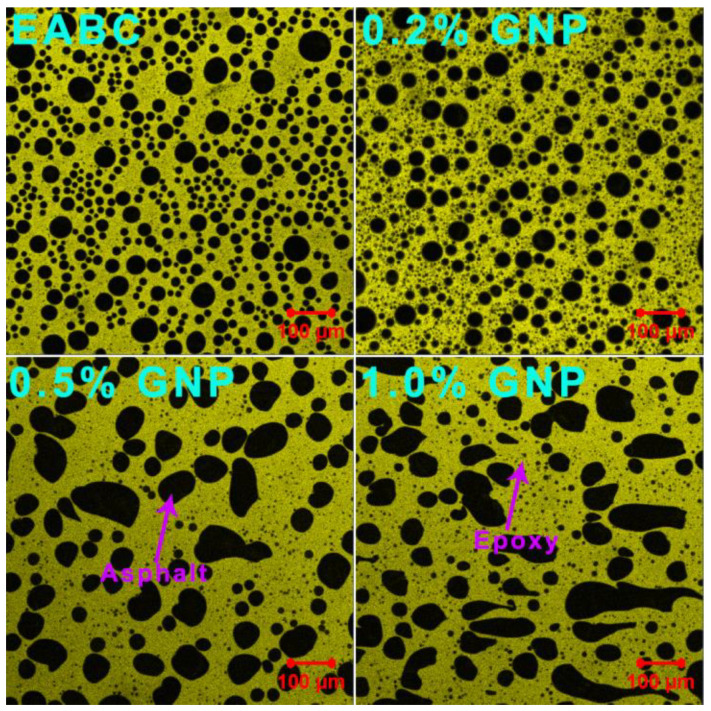
Fluorescence confocal microscopy images of GNP-modified EABCs.

Laser confocal microscopy is a powerful tool for the investigation of the phase separation of epoxy asphalts due to the high contrast resolution between asphalt and crosslinked epoxy resin [31,44]. Figure 2 illustrates the microscopy images of GNP-reinforced EABCs in the fluorescence mode. As is known, asphalt and uncured epoxy resin, mainly composed of epoxy prepolymer and curing agent, are compatible [45]. However, the crosslinked networks formed during epoxy curing lead to the incompatibility between the crosslinked epoxy and asphalt. In this case, as shown in Figure 2, phase separation occurs, and thus results in the dispersion of spherical asphalt particles (black) in the continuous epoxy phase (yellow). When incorporating 0.2% GNP, the shape of asphalt particles is unchanged. However, as the GNP content increases, the shape of asphalt particles becomes irregular. It is worth mentioning that the dispersion of GNPs in EABCs cannot be observed in all of these fluorescence confocal microscopy images.

**Figure 3:**
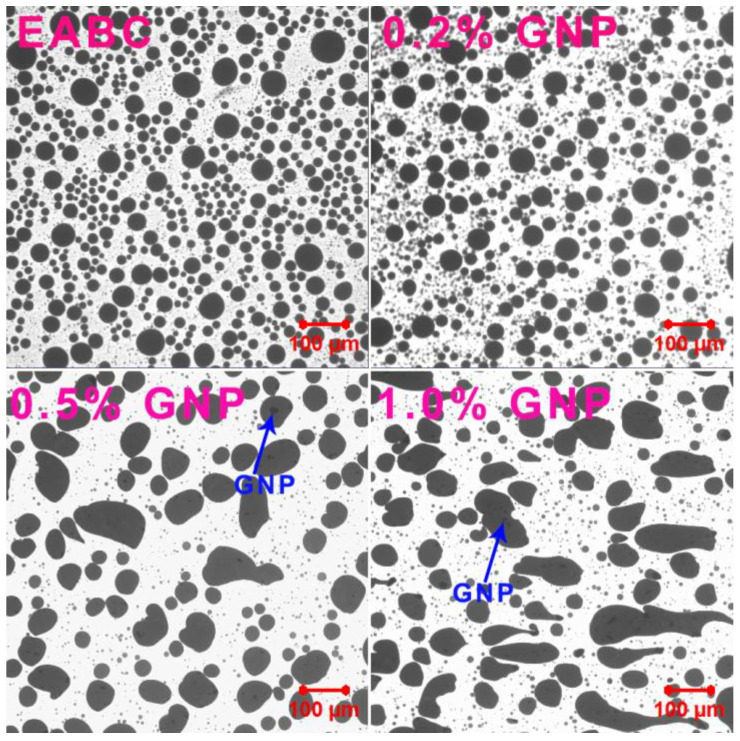
Transmittance confocal microscopy images of GNP-reinforced EABCs.

To distinguish GNPs from both asphalt and epoxy phases of EABCs, transmittance confocal microscopy images were used. As shown in Figure 3, the dispersion of GNPs in the EABC is still unseen. However, darker lamellar GNPs are non-uniformly dispersed in asphalt particles. It is important to note that GNPs are unseen in the epoxy phase, which does not mean that no GNPs exist in the epoxy phase. The lighter color of GNPs with fewer layers may be caused by the invisibility of GNPs in the epoxy phase.

Table 3 collects the *d_n_*, *d_w_* and *PDI*s of the asphalt particles in GNP-reinforced EABCs. The inclusion of GNPs increases the average diameters of asphalt particles of the neat EABC. Besides, the average diameters of asphalt particles of the reinforced EABCs increase in the GNP content. The GNP-reinforced EABCs have a higher *PDI* value than that of the neat EABC, indicating that GNPs lead to the inhomogeneous dispersion of asphalt particles in the continuous epoxy phase. Furthermore, the GNP content has a marginal influence on the dispersion of asphalt particles in the epoxy phase.

### 3.2. Rotational Viscosity

**Figure 4:**
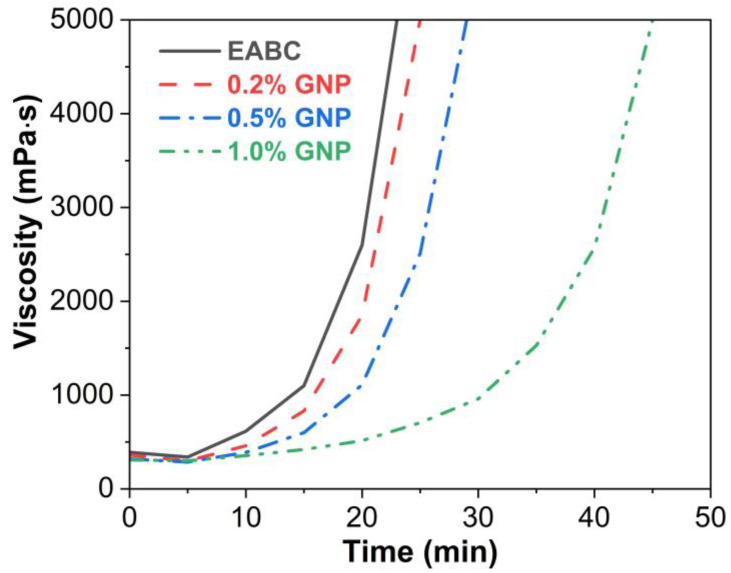
Rotational viscosity as a function of time of GNP-reinforced EABCs at 120 °C.

Figure 4 presents rotational viscosity as a function of time of GNP-reinforced EABCs. At 0 min, the viscosities of the neat EABC and the modified EABCs containing 0.2%, 0.5% and 1.0% GNPs are 390, 360, 320 and 310 mPa·s, respectively, indicating that the viscosity of the neat EABC is declined somewhat with the inclusion of GNPs, which is attributed to the low viscosity of low-molecular-weight curing agent and epoxy prepolymer and the small size of the graphene nanoplatelet, whose thin lamellar structure plays a lubricating role [46]. As time proceeds, the viscosity of all EABCs slightly declines within 5 min, because the reaction between epoxy prepolymers and curing agents is exothermic, and thus results in a typical viscosity decrease [43]. As the reaction proceeds, the viscosity decrease caused by heat absorption is much lower than the viscosity increase generated by the molecular weight increase in epoxy resin. In this case, the viscosity increases gradually with time as the molecular weight increases until the appearance of the gel point. After that, the viscosity increases sharply. Evidently, the viscosity of the neat EABC is higher than that of GNP-reinforced ones during the whole curing process. Furthermore, the viscosity of the reinforced EABCs decreases in the GNP content. In other words, the existence of GNPs delays the curing between epoxy prepolymers and curing agents due to the lubricating effect of the lamellar structure of graphene nanoplatelet. Thus, the addition of GNPs extends the allowable construction time of the neat EABC. Additionally, with the increase in the GNP content, the allowable construction time of the reinforced EABCs is prolonged.

Similar to GNPs, other nanofillers, such as GO, MMT and ATT, also extend the allowable construction time of the neat EABC [35,47,48]. Table 4 summarizes the time to 5000 mPa·s of GNP-reinforced EABCs at 120 °C. Among all of the modified EABCs, GNP-reinforced EABC with 1.0% GNPs exhibits the longest allowable construction time.

### 3.3. Hydrophobicity

**Figure 5:**
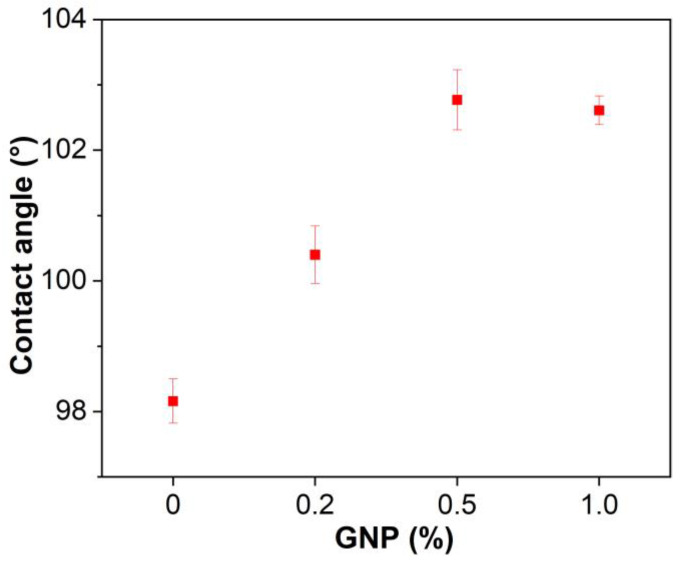
Water contact angles of GNP-reinforced EABCs.

The water contact angles of GNP-reinforced EABCs are presented in Figure 5. The water contact angle of the neat EABC is 98.2°. Clearly, GNPs increase the water contact angle of the neat EABC. The water contact angle of the reinforced EABCs slightly increases with GNP content. A maximum value (102.6°) appears at 0.5%, which is 4.4° higher than that of the neat EABC. The water contact angle (102.3°) slightly decreases when the GNP content increases to 1.0%. It is known that a material is hydrophobic when its water contact angle is over than 90°. Therefore, GNPs improve the hydrophobicity of the neat EABC, which is attributed to the inherently high hydrophobicity of GNPs [49].

### 3.4. Viscoelasticity

#### 3.4.1. Storage Modulus (*E*′)

**Figure 6:**
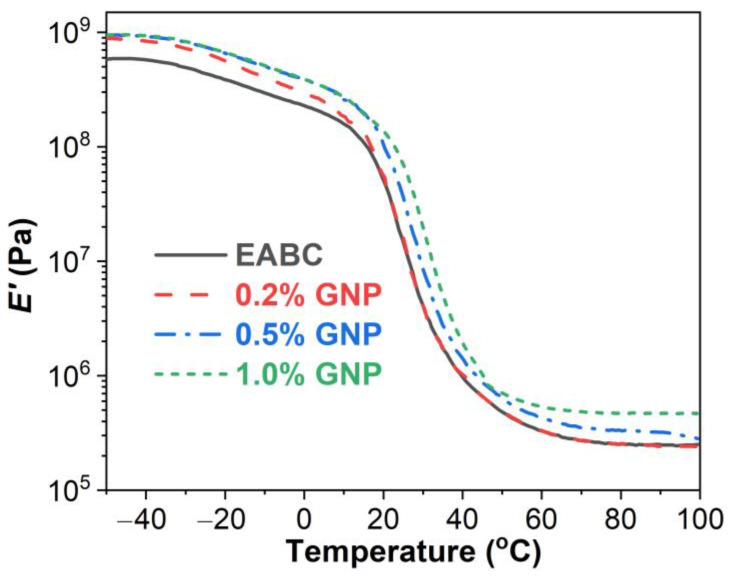
*E*′ as a function of temperature of GNP-reinforced EABCs.

The *E*′ as a function of temperature of GNP-reinforced EABCs is shown in Figure 6. As the temperature increases, the storage modulus gradually decreases at the glassy state (T < *T*_g_, glass transition temperature), followed by a sharp decrease and a gradual decrease during the glass transition and rubbery (T > *T*_g_) stages. When adding 0.2% GNP, the *E*′ of the neat EABC is improved in the glassy state, while the increase in the *E*′ during the glass transition and rubbery stages is imperceptible. When adding more than 0.2% GNPs, the *E*′ of the neat EABC is enhanced over the whole temperature range.

#### 3.4.2. Loss Modulus (*E*″)

Generally, in DMA, the *T*_g_ can either be determined as the inflection point at which a sudden drop of the storage modulus takes place in a storage modulus vs. temperature curve, or the temperature at the maximum *E*″ or loss factor (tan δ) in an *E*″ or tan δ vs. temperature curve [50]. In this case, the *T*_g_ value can be different for a given polymer when being determined using different DMA parameters, because the inflection point of the *E*′ takes place at a temperature lower than the temperature of the maximum *E*″, eventually followed by the temperature of the maximum tan δ. In this work, both *E*″ and tan δ were used to evaluate the *T*_g_s of GNP-reinforced EABCs.

**Figure 7:**
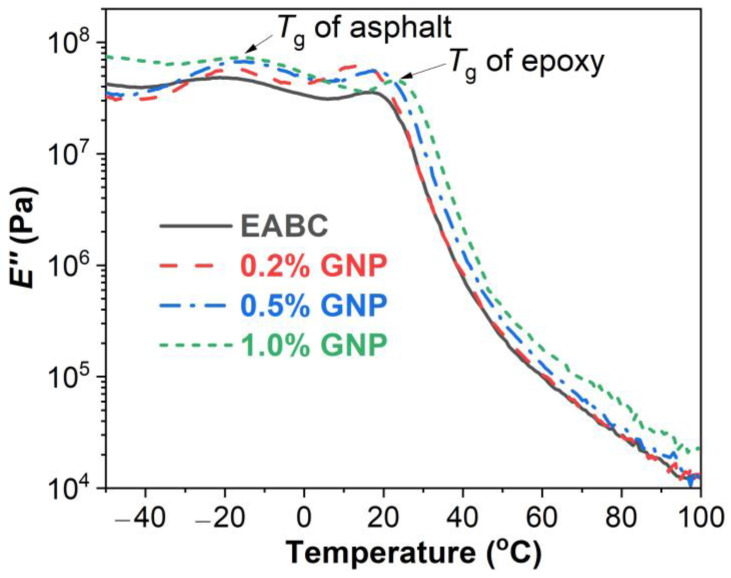
*E*″ as a function of GNP-reinforced EABCs.

*E*″ as a function of temperature of GNP-reinforced EABCs, is depicted in Figure 7. Two peaks appear at the glassy state in all *E*″-versus-temperature curves, which can be attributed to the *T*_g_s of asphalt and of epoxy [51]. From a polymeric material point of view, epoxy asphalt is a polymer blend containing epoxy and asphalt, which have two individual *T*_g_s [52]. Epoxy asphalt (epoxy/asphalt blend) exhibits two distinct *T*_g_s—the *T*_g_ of crosslinked epoxy resin at a higher temperature, and the *T*_g_ of asphalt at a lower temperature—because of the incompatibility between crosslinked epoxy resin and asphalt. Table 5 presents the *T*_g_s of epoxy and asphalt of GNP-reinforced EABCs obtained from loss modulus–temperature curves. The *T*_g_s of asphalt and epoxy of the neat EABC are −21.3 °C and 14.5 °C, respectively. GNPs increase the *T*_g_ of asphalt for the neat EABC. GNPs increase the *T*_g_ of epoxy of the neat EABC apart from the 0.2% loading. Additionally, with the increase in the GNP content, the *T*_g_s of both asphalt and epoxy of the reinforced EABCs increase.

#### 3.4.3. Loss Factor

**Figure 8:**
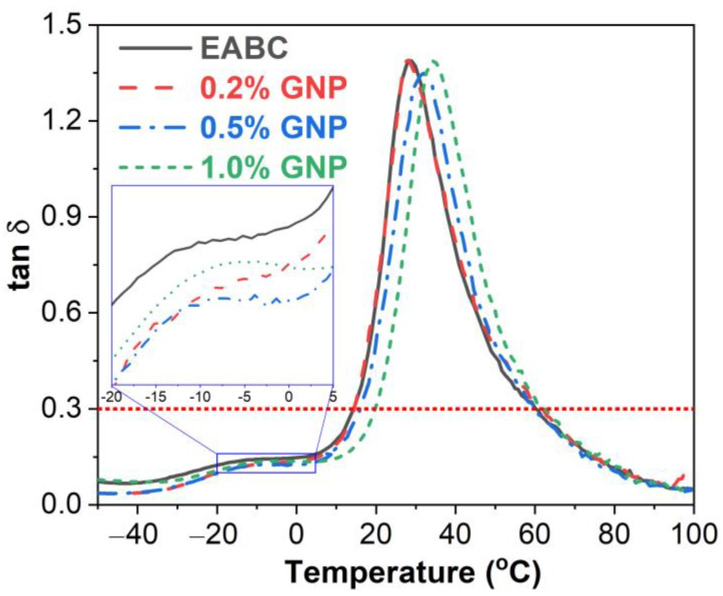
tan δ as a function of temperature of GNP-reinforced EABCs.

The tan δ as a function of temperature of GNP-reinforced EABCs is illustrated in Figure 8. Like loss modulus–temperature curves, two peaks are exhibited in all tan δ–temperature curves. In addition, compared to the tan δ peak of the asphalt, the peak of epoxy is more intensive and broader. The *T*_g_ of asphalt and the *T*_g_ of epoxy of the pure EABC are −12.9 °C and 28.1 °C, respectively, as shown in Table 5. GNPs increase the *T*_g_ of asphalt of the pure EABC. Nevertheless, the GNP content has little influence on the *T*_g_ of asphalt of the reinforced EABCs. When adding 0.2% GNP, the *T*_g_ of epoxy of the neat EABC is nearly unchanged. The *T*_g_s of epoxy of the modified EABCs with more than 0.2% GNP are greater than that of the pure EABC. Furthermore, with increasing GNP content, the glass transition temperature of epoxy of the reinforced EABCs increases.

It is believed that the *T*_g_ of a thermosetting polymer depends greatly on the crosslinking density (*CD*). The crosslinking density can be calculated using Equation (3) based on the elasticity theory [45]:

(3) $$CD=\frac{{E^{\prime }}_{r}}{3RT_{r}}$$

where *E*′*_r_* is the *E*′ at *T_r_* (*T*_g_ of epoxy + 40 K) in the rubbery state. *R* is the gas constant. As can be observed from Table 5, except for the loading of 0.2%, GNPs increase the crosslinking density of the pure EABC. Moreover, the crosslinking density of the reinforced EABCs increases in the GNP content. Consequently, the *T*_g_s of both asphalt and epoxy of the neat EABC are enhanced with the incorporation of GNPs apart from the 0.2% GNP content for the *T*_g_ of epoxy, as discussed previously.

#### 3.4.4. Cole–Cole Plot

**Figure 9:**
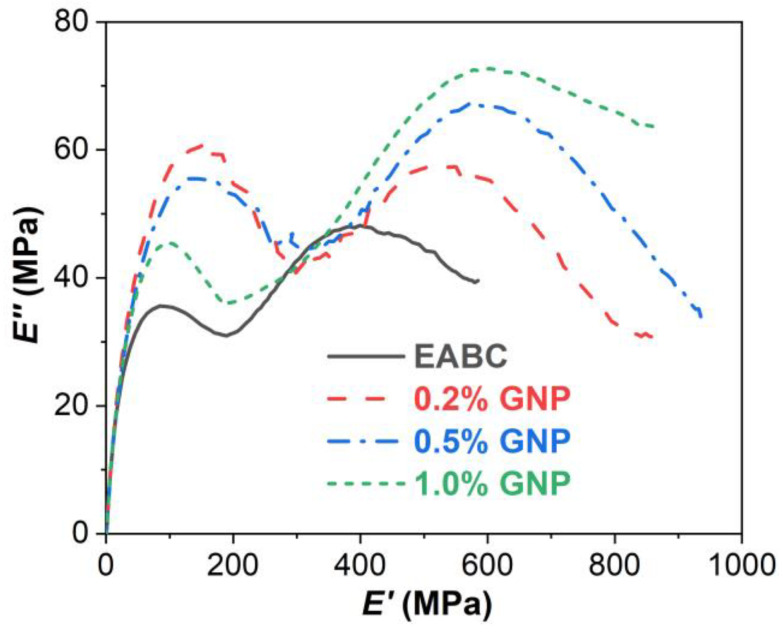
Cole–Cole plots of GNP-reinforced EABCs.

The *E*″-*E*′ curve is called a Cole–Cole plot, and is a useful tool for describing the effect of microfiller and nanofiller on the homogeneous or heterogeneous phase and structural changes in polymer composites [53]. A homogeneous polymeric system shows a smooth semicircular arc, whereas a heterogeneous polymeric system exhibits two or more semicircular arcs. Furthermore, the imperfect or irregular shape of semicircular arcs indicates phase heterogeneity or ununiform dispersion in the polymeric system [54]. The Cole–Cole plots of the GNP-reinforced EABCs are shown in Figure 9. For the neat EABC, there are two smooth semicircular arcs: the one at the higher modulus is the epoxy resin and the other one at the lower modulus is for asphalt and for the epoxy resin because of the incompatibility between the crosslinked epoxy and asphalt. In this case, phase-separated microstructures appear in all EABCs (Figure 2 and Figure 3). With the addition of GNPs, both arcs for asphalt and epoxy shift to the higher modulus because of the reinforcement effect of graphene nanoplatelets. Meanwhile, the shape of the asphalt arcs of the GNP-reinforced EABCs becomes irregular compared to that of epoxy arcs, indicating that the dispersion of GNPs in the asphalt is inhomogeneous as shown in Figure 3.

### 3.5. Mechanical Performance

**Figure 10:**
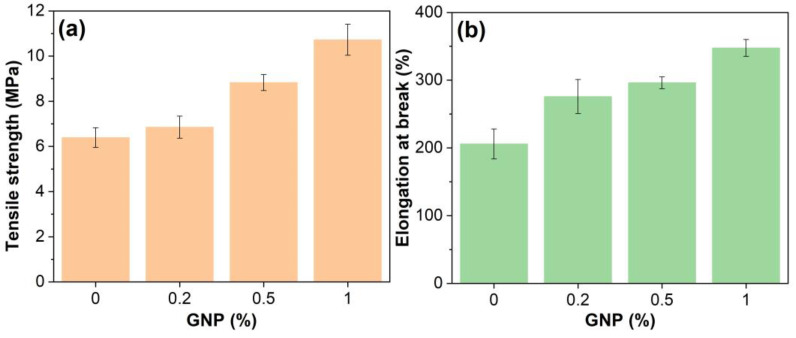
Tensile strength (**a**) and elongation at break (**b**) of GNP-reinforced EABCs.

Figure 10 presents the mechanical properties of GNP-reinforced EABCs. GNPs significantly enhance the mechanical performance of the neat EABC. Furthermore, with increasing GNP content, the mechanical performance of the modified EABCs increases. The elongation at break and tensile strength of the neat EABC increase by 82% and 68%, respectively, with the inclusion of 1.0% GNP. Klimek-McDonald and coworkers revealed that the tensile strength of the neat epoxy is improved when incorporating less than 1.5 vol.% GNP [55]. However, a contrary trend was found in another epoxy system [11]. The increase in tensile strength is due to the higher crosslinking density of the neat EABC increased with the addition of GNPs as mentioned previously. Noteworthily, the elongation at break in all these epoxy systems decreases with the presence of GNPs. As shown in Figure 3, GNPs are dispersed in the dispersed asphalt phase, and thus increase the elongation at break of the pure EABC [56].

**Figure 11:**
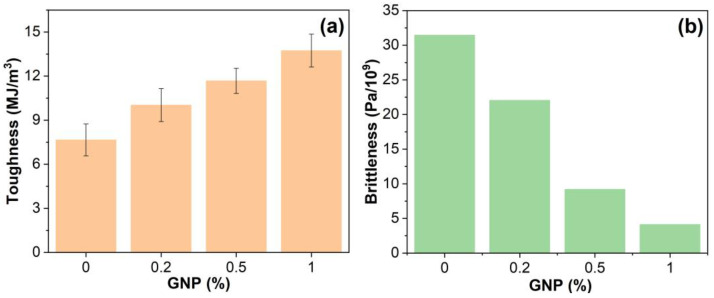
Toughness (**a**) and brittleness (**b**) of GNP-reinforced EABCs.

In a tensile test, the integral of the stress (*σ*) vs. strain (*ε*) curve indicates the toughness (*τ*) of the material [57,58]. Thus,

(4) $$\tau =\int_{0}^{\varepsilon_{b}}\sigma d\varepsilon$$

where *ε_b_* is the elongation at break. To determine the brittleness (*B*) of polymeric materials, Brostow et al. [59] proposed an empirical equation:

(5) $$B=\frac{1}{\varepsilon_{b}E^{\prime }{}_{RT}}$$

where *E*′*_RT_* is the *E*′ at room temperature gained from DMA. The toughness and brittleness of GNP-reinforced EABCs are illustrated in Figure 11. GNPs improve the toughness and thus decrease the brittleness of the neat EABC. Additionally, with the increase in the GNP content, the toughness of the reinforced EABCs increases (Figure 11a). Correspondingly, the brittleness of the modified EABCs exhibits an opposite trend (Figure 11b). With the incorporation of 1.0% GNP, the toughness of the pure EABC increases by 79%.

### 3.6. Bonding Properties

#### 3.6.1. Single-Lap Shear Strength

**Figure 12:**
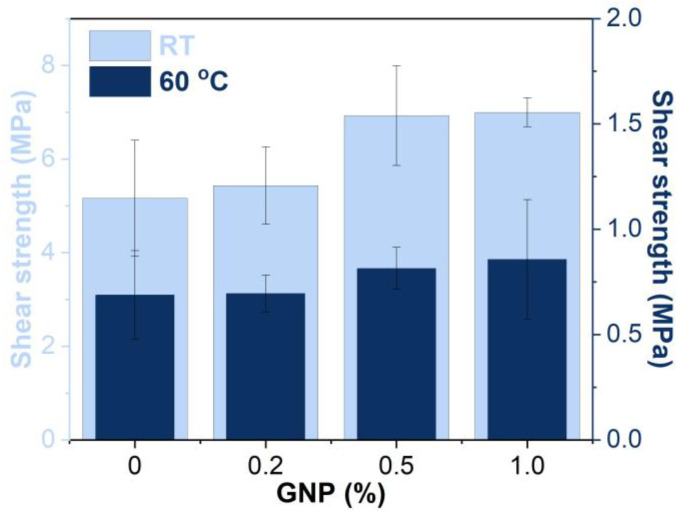
Shear strengths of GNP-reinforced EABCs at room temperature and 60 °C.

The shear strengths of GNP-reinforced EABCs at room temperature and 60 °C are shown in Figure 12. GNPs enhance the shear strength of the neat EABC. Furthermore, with the increase in GNP content, the shear strength of the reinforced EABCs increases. When adding 1.0% GNP, the shear strengths of the neat EABC at 60 °C and room temperature increase by 26% and 36%, respectively.

**Figure 13:**
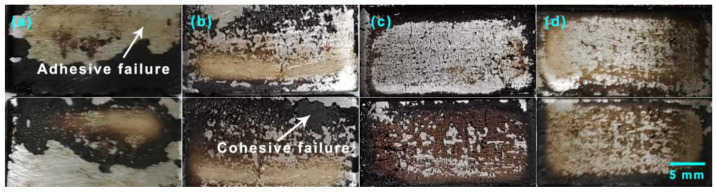
Failure surfaces of steel plates bonded with GNP-reinforced EABCs: EABC (**a**), 0.2% (**b**), 0.5% (**c**) and 1.0% (**d**) after single-lap shear tests at room temperature.

**Figure 14:**
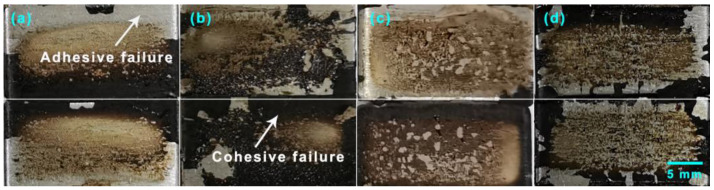
Failure surfaces of steel plates bonded with GNP-reinforced EABCs: EABC (**a**), 0.2% (**b**), 0.5% (**c**) and 1.0% (**d**) after single-lap shear tests at 60 °C.

The failure surfaces of GNP-reinforced EABCs after single-lap shear tests are shown in Figure 13 and Figure 14. The failure surfaces of steel plates exhibit a mixed mode of cohesive and adhesive failures. Generally, adhesive failure takes place at the interface of the bond coat and the substrate. The bonding layer at the interface weakens and leads to failure with the smooth surface left on the substrate, which exhibits weak bonding strength. Cohesive failure occurs within the bond coat with the rough surface left on the substrate, indicating that the bonding strength of the bond coat itself is lower than the bonding strength between the bond coat and the substrate. In this case, cohesive failure reveals good bonding strength [60]. For a bond coat with a mixed failure mode, the lower the area of adhesive failure is, the higher the bonding strength is.

**Figure 15:**
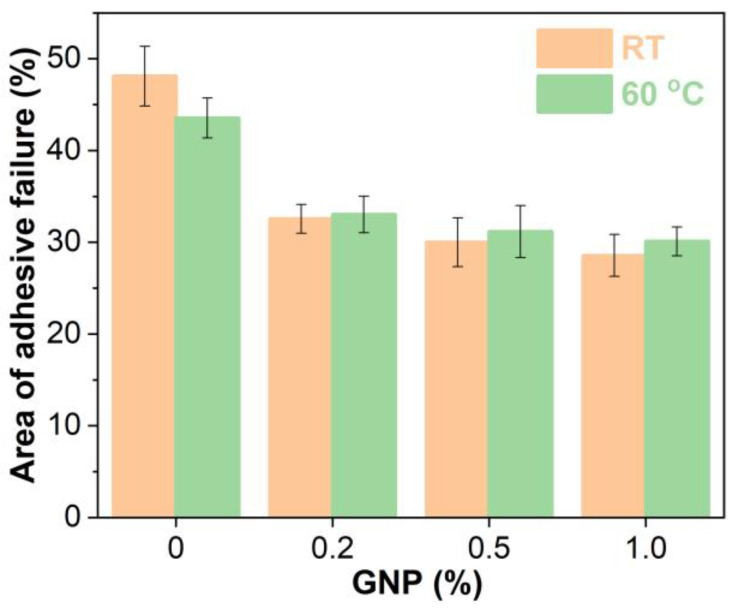
Area of adhesive failure on the steel plates bonded with GNP-reinforced EABCs after single-lap shear tests.

The areas of adhesive failure on the steel plates bonded with GNP-reinforced EABCs after single-lap shear tests are illustrated in Figure 15. For the neat EABC, the area of adhesive failure and cohesive failure at room temperature are nearly the same. However, at 60 °C, the area of adhesive failure is slightly lower than that of cohesive failure. When adding GNPs, cohesive failure mode becomes dominant, and thus the presence of GNPs enhances the shear strength of the neat EABC. Furthermore, with increasing GNP content, the area of adhesive failure of the reinforced EABCs slightly decreases. Consequently, for the reinforced EABCs, the shear strength increases as the GNP content increases.

#### 3.6.2. Pull-Off Adhesion Strength

**Figure 16:**
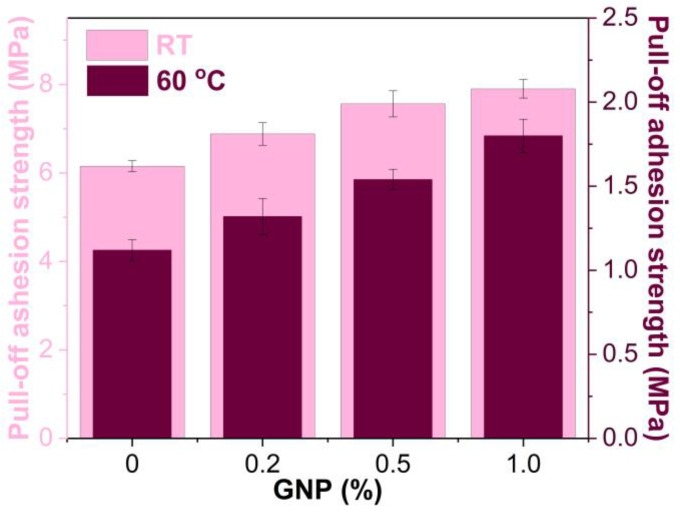
Pull-off adhesion strengths of GNP-reinforced EABCs at room temperature and 60 °C.

The pull-off adhesion strengths of GNP-reinforced EABCs at room temperature and 60 °C are shown in Figure 16. Like single-lap shear strength, the pull-off adhesion strength of the neat EABC is enhanced with the incorporation of GNPs. For the modified EABCs, the pull-off adhesion strength increases with the GNP content. When adding 1.0% GNP, the pull-off adhesion strengths of the pure EABC at 60 °C and room temperature increase by 61% and 28%, respectively.

**Figure 17:**
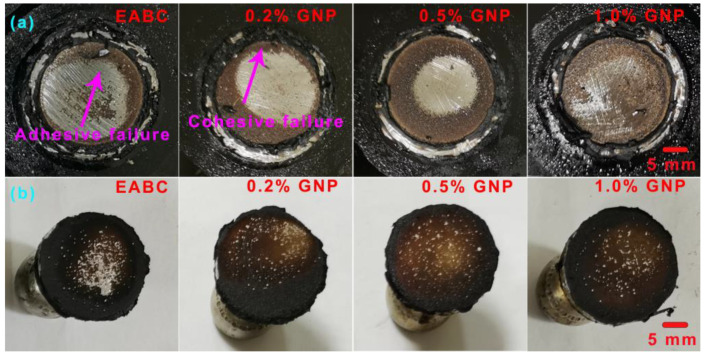
Surfaces of steel plates (**a**) and dollies (**b**) bonded with GNP-reinforced EABCs after room-temperature pull-off adhesion tests.

**Figure 18:**
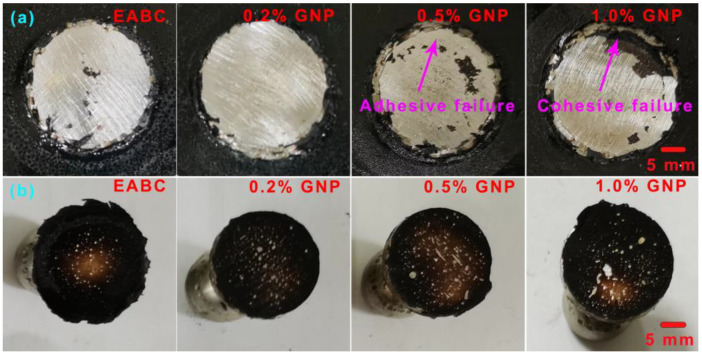
Surfaces of steel plates (**a**) and dollies (**b**) bonded by GNP-reinforced EABCs after pull-off adhesion tests at 60 °C.

Figure 17 and Figure 18 present the surfaces of steel plates and dollies bonded with GNP-reinforced EABCs after pull-off adhesion tests. For the dollies, adhesive failure is overwhelming both at room temperature and 60 °C. For steel plates, the room-temperature pull-off failure mode is a mixed failure. However, adhesive failure is overwhelming at 60 °C.

**Figure 19:**
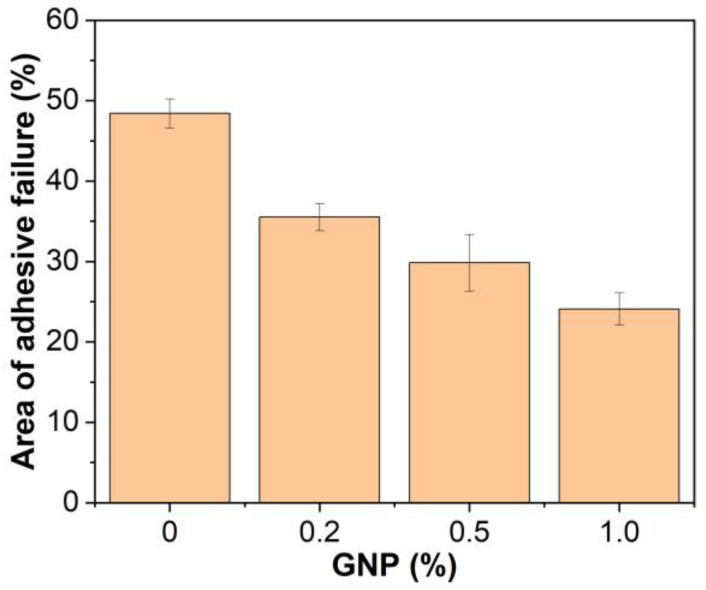
Area of adhesive failure of steel plates bonded by GNP-reinforced EABCs after room-temperature pull-off adhesion tests.

Figure 19 shows the areas of adhesive failure of steel plates bonded by GNP-reinforced EABCs after room-temperature pull-off adhesion tests. Similar to single-lap shear tests, the areas of adhesive failure and cohesive failure of the neat EABC are nearly equivalent. When incorporating GNPs, the area of adhesive failure of the neat EABC significantly decreases. Besides, in the case of GNP reinforced EABCs, the area of adhesive failure decreases with the GNP content. Thus, GNPs enhance the pull-off adhesion strength of the neat EABC. Moreover, with the increase in the GNP content, the pull-off strength of reinforced EABCs increases. It is known that, in addition to high mechanical strength and modulus, graphene materials have a high aspect ratio, which increases the load transfer to the polymer matrix and thus improves the bonding strength of the neat EABC [61].

## 4. Conclusions

In this study, GNP-reinforced EABCs were prepared and characterized. When adding more than 0.2% GNP, the shape of spherical asphalt particles in EABCs becomes irregular. GNPs increas the diameters of the neat EABC. Furthermore, with the increase in GNP content, the average diameters of the reinforced EABCs increase. Laser confocal microscopy observation revealed that GNPs are dispersed in the dispersed asphalt phase of the EABC. Due to the lubricating effect of the GNPs, the viscosity of the pure EABC is lowered, indicating that the allowable construction time of the neat EABC is extended with the inclusion of GNPs. In addition, the extension effect is more evident with higher GNP content. GNPs improve the hydrophobicity of the neat EABC. When introducing over 0.2% GNP, the storage modulus during the whole temperature range, the crosslinking density and the *T*_g_ of the epoxy and the *T*_g_ of asphalt are increased. Cole–Cole plots revealed that GNPs result in the inhomogeneity of asphalt and thus the non-uniform dispersion of graphene nanoplatelets in the asphalt particles of the EABC. GNPs significantly improved both the mechanical and bonding properties of the neat EABC. Furthermore, with increasing GNP content, the mechanical and bonding strengths of the reinforced EABCs increased. When adding 1.0% GNP, the tensile strength, elongation at break and toughness of the neat EABC increased by 68%, 82% and 79%, respectively. The shear strengths and pull-off adhesion strengths of the neat EABC at room temperature and 60 °C improved by 36%, 28%, 26% and 61%, respectively.

## Figures and Tables

**Table 1:** Property of graphene oxide.

Property	Value
Number of layers	5~10
Diameter (µm)	5~50
Thickness (nm)	3.5~8
Purity (%)	95

**Table 2:** Overview of base asphalt.

Property	Standard	Value
Penetration (25 °C, 0.1 mm)	ASTM D5-06	91.0
Ductility (10 °C, cm)	ASTM D113-07	93.0
Softening point (°C)	ASTM D36-06	46.3
Viscosity (120 °C, mPa·s)	ASTM D4402-06	787
Saturates (%)	ASTM D4124-09	16.7
Aromatics (%)		33.9
Resins (%)		44.7
Asphaltenes (%)		4.7

**Table 3:** *d_n_*, *d_w_* and *PDI*s of the asphalt particles in GNP-reinforced EABCs.

GNP (%)	*d_n_* (mm)	*d_w_* (mm)	*PDI*
0	18.6 ± 1.6	21.1 ± 1.8	1.13 ± 0.01
0.2	21.6 ± 1.7	26.2 ± 3.8	1.21 ± 0.09
0.5	26.0 ± 6.5	32.0 ± 4.7	1.27 ± 0.16
1.0	32.4 ± 3.4	40.5 ± 5.9	1.25 ± 0.04

**Table 4:** Time to 5000 mPa·s of GNP-reinforced EABCs at 120 °C.

Nanofiller	Time to 5000 mPa·s (Minute)				Reference
	0	0.2%	0.5%	1.0%	
GNP	23	25	29	45	This work
GO	23	25	27	30	[35]
MMT	23	-	-	26	[47]
ATT	23	-	-	36	[48]

**Table 5:** *T*_g_s of epoxy and asphalt and crosslink densities for GNP-reinforced EABCs.

GNP (%)	*T*_g_ of Asphalt (°C)		*T*_g_ of Epoxy (°C)		*CD* (mol/m^3^)
	tan δ	*E*″	tan δ	*E*″	
0	−12.9	−21.3	28.1	14.5	32.8
0.2	−9.1	−18.8	27.8	12.9	33.2
0.5	−8.8	−14.7	31.9	18.8	40.2
1.0	−9.3	−14.3	34.4	23.3	54.7

## Data Availability

All data are available in the manuscript.

## References

[1] Hu K., Kulkarni D.D., Choi I., Tsukruk V.V. (2014). Graphene-polymer nanocomposites for structural and functional applications. Prog. Polym. Sci..

[2] Geim A.K. (2009). Graphene: Status and prospects. Science.

[3] Tiwari S.K., Sahoo S., Wang N., Huczko A. (2020). Graphene research and their outputs: Status and prospect. J. Sci. Adv. Mater. Devices.

[4] Shi G., Araby S., Gibson C.T., Meng Q., Zhu S., Ma J. (2018). Graphene platelets and their polymer composites: Fabrication, structure, properties, and applications. Adv. Funct. Mater..

[5] Zhao R., Jing F., Li C., Wang R., Xi Z., Cai J., Wang Q., Xie H. (2022). Viscosity-curing time behavior, viscoelastic properties, and phase separation of graphene oxide/epoxy asphalt composites. Polym. Compos..

[6] Botta L., Scaffaro R., Sutera F., Mistretta M.C. (2018). Reprocessing of PLA/graphene nanoplatelets nanocomposites. Polymers.

[7] Gaska K., Xu X., Gubanski S., Kádár R. (2017). Electrical, mechanical, and thermal properties of ldpe graphene nanoplatelets composites produced by means of melt extrusion process. Polymers.

[8] Mostovoy A., Shcherbakov A., Yakovlev A., Arzamastsev S., Lopukhova M. (2022). Reinforced epoxy composites modified with functionalized graphene oxide. Polymers.

[9] Le M.-T., Huang S.-C. (2015). Thermal and mechanical behavior of hybrid polymer nanocomposite reinforced with graphene nanoplatelets. Materials.

[10] Palomba M., Carotenuto G., Longo A., Sorrentino A., Di Bartolomeo A., Iemmo L., Urban F., Giubileo F., Barucca G., Rovere M. (2019). Thermoresistive properties of graphite platelet films supported by different substrates. Materials.

[11] King J.A., Klimek D.R., Miskioglu I., Odegard G.M. (2013). Mechanical properties of graphene nanoplatelet/epoxy composites. J. Appl. Polym. Sci..

[12] Panta J., Zhang Y.X., Rider A.N., Wang J. (2022). Ozone functionalized graphene nanoplatelets and triblock copolymer hybrids as nanoscale modifiers to enhance the mechanical performance of epoxy adhesives. Int. J. Adhes. Adhes..

[13] Zhang G., Wang F., Dai J., Huang Z. (2016). Effect of functionalization of graphene nanoplatelets on the mechanical and thermal properties of silicone rubber composites. Materials.

[14] Cataldi P., Athanassiou A., Bayer I.S. (2018). Graphene nanoplatelets-based advanced materials and recent progress in sustainable applications. Appl. Sci..

[15] Gorrasi G., Bugatti V., Milone C., Mastronardo E., Piperopoulos E., Iemmo L., Di Bartolomeo A. (2018). Effect of temperature and morphology on the electrical properties of PET/conductive nanofillers composites. Compos. Part B.

[16] Chieng B.W., Ibrahim N.A., Yunus W.M.Z.W., Hussein M.Z. (2014). Poly(lactic acid)/poly(ethylene glycol) polymer nanocomposites: Effects of graphene nanoplatelets. Polymers.

[17] Pereira P., Ferreira D.P., Araújo J.C., Ferreira A., Fangueiro R. (2020). The potential of graphene nanoplatelets in the development of smart and multifunctional ecocomposites. Polymers.

[18] Marra F., D’Aloia A.G., Tamburrano A., Ochando I.M., De Bellis G., Ellis G., Sarto M.S. (2016). Electromagnetic and dynamic mechanical properties of epoxy and vinylester-based composites filled with graphene nanoplatelets. Polymers.

[19] Ali I., Kim N.K., Bhattacharyya D. (2021). Effects of graphene nanoplatelets on mechanical and fire performance of flax polypropylene composites with intumescent flame retardant. Molecules.

[20] Mangus A., Chen W.-F., Duan L. (2014). Orthotropic steel decks. Bridge Engineering Handbook: Superstructure Design.

[21] Bocci E., Canestrari F. (2012). Analysis of structural compatibility at interface between asphalt concrete pavements and orthotropic steel deck surfaces. Transp. Res. Rec..

[22] Huang Q., Qian Z., Chen L., Zhang M., Zhang X., Sun J., Hu J. (2020). Evaluation of epoxy asphalt rubber with silane coupling agent used as tack coat for seasonally frozen orthotropic steel bridge decks. Constr. Build. Mater..

[23] Cuadri A.A., Delgado-Sánchez C., Navarro F.J., Partal P. (2020). Short- and long-term epoxy modification of bitumen: Modification kinetics, rheological properties, and microstructure. Polymers.

[24] Luo S., Sun J., Hu J., Liu S. (2022). Performance evolution mechanism of hot-mix epoxy asphalt binder and mixture based on component characteristics. J. Mater. Civ. Eng..

[25] Simpson W.C., Sommer H.J., Griffin R.L., Miles T.K. (1960). Epoxy asphalt concrete for airfield pavements. J. Air Transp. Div..

[26] Li M., Min Z., Wang Q., Huang W., Shi Z. (2022). Influence of curing agent ratio, asphalt content and crosslinking degree on the compatibility and component distribution of epoxy asphalt in compound curing agent system. Int. J. Pavement Eng..

[27] Kang Y., Jin R., Wu Q., Pu L., Song M., Cheng J., Yu P. (2016). Anhydrides-cured bimodal rubber-like epoxy asphalt composites: From thermosetting to quasi-thermosetting. Polymers.

[28] Sun J., Huang W., Lu G., Luo S., Li Y. (2023). Investigation of the performance and micro-evolution mechanism of low-content thermosetting epoxy asphalt binder towards sustainable highway and bridge decks paving. J. Clean. Prod..

[29] Wang C., Wu Q., Liu Y., Zhang M., Zhang Z., Zhou D., Kang Y. (2022). Mechanical properties of tri-modal epoxy asphalt composites. Int. J. Pavement Eng..

[30] Xie H., Li C., Wang Q. (2022). A critical review on performance and phase separation of thermosetting epoxy asphalt binders and bond coats. Constr. Build. Mater..

[31] Pipintakos G., Hasheminejad N., Lommaert C., Bocharova A., Blom J. (2021). Application of Atomic Force (AFM), Environmental Scanning Electron (ESEM) and Confocal Laser Scanning Microscopy (CLSM) in bitumen: A review of the ageing effect. Micron.

[32] Zhang Z., Sun J., Huang Z., Wang F., Jia M., Lv W., Ye J. (2021). A laboratory study of epoxy/polyurethane modified asphalt binders and mixtures suitable for flexible bridge deck pavement. Constr. Build. Mater..

[33] Liu Q., Wang C., Zhang Z., Du C., Liu P., Oeser M. (2021). Influence of preparation methods on the performance of cold-mixed epoxy bitumen. Mater. Struct..

[34] Liu Y., Qian Z., Wang Y., Xue Y. (2021). Development and laboratory evaluation of a cold mix high-early-strength epoxy asphalt concrete for steel bridge deck pavements. Materials.

[35] Zhang J., Wang R., Zhao R., Jing F., Li C., Wang Q., Xie H. (2022). Graphene oxide-modified epoxy asphalt bond coats with enhanced bonding properties. Materials.

[36] Si J., Wang J., Li Y., Ma J., Ruan W., Yu X., Jiang R. (2022). Enhanced mechanical performances of epoxy asphalt adhesives modified by graphene oxide. Road Mater. Pavement Des..

[37] Anwar Z., Kausar A., Rafique I., Muhammad B. (2016). Advances in epoxy/graphene nanoplatelet composite with enhanced physical properties: A review. Polym.-Plast. Technol. Eng..

[38] Kausar A., Anwar Z., Muhammad B. (2016). Recent developments in epoxy/graphite, epoxy/graphene, and epoxy/graphene nanoplatelet composites: A comparative review. Polym.-Plast. Technol. Eng..

[39] Moretti L., Fabrizi N., Fiore N., D’Andrea A. (2021). Mechanical characteristics of graphene nanoplatelets-modified asphalt mixes: A comparison with polymer- and not-modified asphalt mixes. Materials.

[40] Han M., Muhammad Y., Wei Y., Zhu Z., Huang J., Li J. (2021). A review on the development and application of graphene based materials for the fabrication of modified asphalt and cement. Constr. Build. Mater..

[41] Zhang F., Liu X., Zhang L., Zhou S., Huang K. (2023). Preparation and properties of epoxy asphalt modified by biomimetic graphene oxide nanocomposites. J. Mater. Civ. Eng..

[42] Zhang L., Zhang F., Huang K., Zhou S., Guo Y. (2021). Preparation and performance of graphene nanoplatelets-modified epoxy asphalt. J. Perform. Constr. Facil..

[43] Zhao R., Jing F., Li C., Wang R., Xi Z., Cai J., Wang Q., Xie H. (2022). Phase-separated microstructures and viscosity-time behavior of graphene nanoplatelet modified warm-mix epoxy asphalt binders. Mater. Struct..

[44] Gong J., Jing F., Zhao R., Li C., Cai J., Wang Q., Xie H. (2022). Waste cooking oil-modified epoxy asphalt rubber binders with improved compatibility and extended allowable construction time. Molecules.

[45] Zhao R., Jing F., Wang R., Cai J., Zhang J., Wang Q., Xie H. (2022). Influence of oligomer content on viscosity and dynamic mechanical properties of epoxy asphalt binders. Constr. Build. Mater..

[46] Fan Y.H., Yu S.W., Wang H.M., Yao Y.H., Wang Y., Wang C.H. (2019). Study on preparation and properties of graphene reinforced epoxy resin composites. IOP Conf. Ser. Mater. Sci. Eng..

[47] Sun Y., Han X., Su W., Gong J., Xi Z., Zhang J., Wang Q., Xie H. (2020). Mechanical and bonding properties of pristine montmorillonite reinforced epoxy asphalt bond coats. Polym. Compos..

[48] Sun Y., Liu Y., Jiang Y., Xu K., Xi Z., Xie H. (2018). Thermal and mechanical properties of natural fibrous nanoclay reinforced epoxy asphalt adhesives. Int. J. Adhes. Adhes..

[49] Zhang M., Ma Y., Zhu Y., Che J., Xiao Y. (2013). Two-dimensional transparent hydrophobic coating based on liquid-phase exfoliated graphene fluoride. Carbon.

[50] Li G., Lee-Sullivan P., Thring R.W. (2000). Determination of activation energy for glass transition of an epoxy adhesive using dynamic mechanical analysis. J. Therm. Anal. Calorim..

[51] Sun Y., Liu Y., Gong J., Han X., Xi Z., Zhang J., Wang Q., Xie H. (2022). Thermal and bonding properties of epoxy asphalt bond coats. J. Therm. Anal. Calorim..

[52] Turner T.F., Branthaver J.F., Usmani A. (1997). DSC studies of asphalts and asphalt components. Asphalt Science and Technology.

[53] Devi L.U., Bhagawan S.S., Thomas S. (2010). Dynamic mechanical analysis of pineapple leaf/glass hybrid fiber reinforced polyester composites. Polym. Compos..

[54] Saba N., Paridah M.T., Abdan K., Ibrahim N.A. (2016). Dynamic mechanical properties of oil palm nano filler/kenaf/epoxy hybrid nanocomposites. Constr. Build. Mater..

[55] Klimek-McDonald D.R., King J.A., Miskioglu I., Pineda E.J., Odegard G.M. (2018). Determination and modeling of mechanical properties for graphene nanoplatelet/epoxy composites. Polym. Compos..

[56] Han M., Li J., Muhammad Y., Hou D., Zhang F., Yin Y., Duan S. (2018). Effect of polystyrene grafted graphene nanoplatelets on the physical and chemical properties of asphalt binder. Constr. Build. Mater..

[57] Brostow W., Hagg Lobland H.E., Khoja S. (2015). Brittleness and toughness of polymers and other materials. Mater. Lett..

[58] Jing F., Zhao R., Li C., Xi Z., Wang Q., Xie H. (2022). Influence of the epoxy/acid stoichiometry on the cure behavior and mechanical properties of epoxy vitrimers. Molecules.

[59] Brostow W., Hagg Lobland H.E., Narkis M. (2006). Sliding wear, viscoelasticity, and brittleness of polymers. J. Mater. Res..

[60] Kagalkar N., Srinivas S., Dhananjaya B.R. (2018). Determination of shear strength and failure type of the sealant using lap shear test. Mater. Today Proc..

[61] Shim G.H., Kweon B., Lee M.S., Kim J.H., Jun T.S., Kim T., Jerng D.-W., Mahian O., Wongwises S., Ahn H.S. (2021). Highly improved mechanical and thermal properties of alkali silicate and graphene nanoplatelet composite adhesives. Int. J. Adhes. Adhes..

